# Multiple Method Contraception Use among African American Adolescents in Four US Cities

**DOI:** 10.1155/2011/765917

**Published:** 2011-07-18

**Authors:** Jennifer L. Brown, Michael Hennessy, Jessica M. Sales, Ralph J. DiClemente, Laura F. Salazar, Peter A. Vanable, Michael P. Carey, Daniel Romer, Robert F. Valois, Larry K. Brown, Bonita Stanton

**Affiliations:** ^1^Department of Behavioral Sciences & Health Education, Rollins School of Public Health, Emory University, Atlanta, GA 30322, USA; ^2^Annenberg Public Policy Center, University of Pennsylvania, Philadelphia, PA 19104, USA; ^3^Center for Health and Behavior, Syracuse University, Syracuse, NY 13244-5040, USA; ^4^Arnold School of Public Health, University of South Carolina, Columbia, SC 29208, USA; ^5^Department of Psychiatry, Rhode Island Hospital, Brown University, Providence, RI 02912, USA; ^6^Department of Pediatrics, Wayne State University, Detroit, MI 48201-2196, USA

## Abstract

We report on African American adolescents' (*N* = 850; *M* age = 15.4) contraceptive practices and type of contraception utilized during their last sexual encounter. Respondents completed measures of demographics, contraceptive use, sexual partner type, and ability to select “safe” sexual partners. 40% endorsed use of dual or multiple contraceptive methods; a total of 35 different contraceptive combinations were reported. Perceived ability to select “safe” partners was associated with not using contraception (OR = 1.25), using less effective contraceptive methods (OR = 1.23), or hormonal birth control (OR = 1.50). Female gender predicted hormonal birth control use (OR = 2.33), use of less effective contraceptive methods (e.g., withdrawal; OR = 2.47), and using no contraception (OR = 2.37). Respondents' age and partner type did not predict contraception use. Adolescents used contraceptive methods with limited ability to prevent both unintended pregnancies and STD/HIV. Adolescents who believed their partners posed low risk were more likely to use contraceptive practices other than condoms or no contraception. Reproductive health practitioners are encouraged to help youth negotiate contraceptive use with partners, regardless of the partner's perceived riskiness.

## 1. Introduction

A sizeable minority of adolescents experience unintended pregnancies and sexually transmitted diseases (STDs) [1, 2]. Unintended pregnancy rates are the highest among individuals less than 20 years of age, despite availability of effective contraceptive methods [2]. STD rates are particularly concerning for young African American adolescents [3] because national estimates show that African American youth between the ages of 15 and 24 experience the highest rates of chlamydia and gonorrhea [3, 4], and these STD increase susceptibility to HIV [5–7].

Clinical adolescent medicine guidelines recommend comprehensive reproductive health counseling to encourage use of a “dual method” strategy to prevent pregnancy through hormonal birth control use and STD/HIV through consistent use of male latex condoms [8, 9]. However, research suggests that use of hormonal birth control use is associated with decreased condom use [10, 11], and, despite being the recommended practice, rates of reported dual method use among adolescents are low with usage rates ranging from 5.5% to 14.6% [8, 12–14]. The prevalence of dual method use among African American adolescents is uncertain. A nationally representative sample of adolescents indicated that dual method use was significantly lower among African American youth [8]. However, other studies have suggested that dual method use may be more prevalent among African American adolescents [13, 14].

Few studies have examined factors associated with African American adolescents' selection and use of dual method protection strategies [13–16]. Further, most studies have focused on African American female adolescents' use of dual methods with only one study including males [15]. Selection of contraceptive methods and use of dual methods may vary based on the type of partner and characteristics of the relationship. For example, a qualitative study found that the type of sexual partner influenced dual method use; dual use of condoms and hormonal birth control was less likely in relationships with steady sexual partners [17]. Another study identified that consistent use of hormonal birth control as the only contraception method was more likely with steady sexual partners [18]. Thus, evidence suggests that adolescents may vary their contraception method use by partner type. In addition, adolescents' perceived ability to identify “risky” sexual partners may impact contraception selection and use. Indeed, lower condom use has been linked to perceiving a partner as “safe” or presenting low risk for STD/HIV transmission [19, 20]. 

To address gaps in the existing research, this study examines the contraception methods used by African-American adolescents in four US cities. This study examines the influence of partner type and perceived ability to select safe sexual partners in relationship to contraception use. In addition, a large sample of African American adolescent females *and *males was recruited, allowing us to examine whether contraception practices differ by gender or age. We describe the types and combinations of contraception methods used during respondents' last sexual encounter and predict adolescents' use of contraception method types based on their gender, age, pregnancy history, perceived ability to select safer sexual partners, and the type of sexual partner.

## 2. Methods

### 2.1. Study Design

Project iMPPACS was a longitudinal intervention project for African American adolescents living in low-income urban areas designed to evaluate the effect of community-wide media campaigns to supplement and reinforce (i.e., act as “booster sessions”) small group interventions to increase condom use and reduce sexual risk-taking. The media boosters were implemented to prevent decay of the small group intervention's impact to lower HIV and STI risk over time [21–23]. The design of Project iMPPACS was a 2 (sexual risk reduction or a general health promotion intervention) by 2 (media present or media absent) by 6 (time: baseline, 3, 6, 12, 18, and 36 months after-recruitment) randomized controlled trial implemented in two northern cities (Providence, RI, and Syracuse, NY) and two southern cities (Columbia, SC, and Macon, Ga). Once recruited, consented, and assented, adolescents completed a baseline audio computer-assisted self-interview to assess their sexual attitudes, beliefs, and sexual behaviors. Analyses reported in this paper are limited to baseline data collected prior to participation in the small group intervention sessions. Additional information on implementation of Project iMPPACS can be found elsewhere [24]. All study procedures were approved by the Institutional Review Boards at the affiliated study institutions.

### 2.2. Measures

#### 2.2.1. Demographics

Demographic characteristics assessed were age (14-15 versus 16-17 at baseline) and gender.

#### 2.2.2. Contraceptive Use during Last Sexual Encounter

The contraceptive behavior item was only asked of respondents reporting at least one lifetime event of vaginal sex (*N* = 850). The item stem was “The last time you had vaginal sex, what method did you or your partner use to prevent pregnancy? (check all that apply).” The responses were (1) no method was used to prevent pregnancy, (2) birth control pills, (3) condoms (rubber), (4) Depo-Provera (injectable birth control), (5) had sex during a safe time of the month (rhythm method), (6) pulled out before ejaculating (cumming)/withdrawal, and (7) some other methods.

#### 2.2.3. Sexual Partner Type during the Last Sexual Encounter

Research shows differences in regularity and type of contraceptive use by partner type among adolescents and adults [25–28]. Thus, respondents characterized their sexual partner during the most recent sexual encounter as one of three possible partner types: (1) someone you just met or a casual friend, (2) someone you knew well, but not a regular or “steady” partner, and (3) a steady boyfriend or girlfriend.

#### 2.2.4. Thematic Mediator of Condom Use

Behavioral change is produced through modification of the causal mediating variables [29, 30]. For the analyses reported in this paper, we used a measure of one of the three mass media themes directly related to condom use: that a person can select a “safe sexual partner.” We label this as the *Select *variable [31] and constructed it from three items in the Condom Attitude Scale [32]: “A condom is not necessary if you are pretty sure the other person does not have a sexually transmitted disease,” “A condom is not necessary if you know your partners,” and “A condom is not necessary when you and your partner agree not to have sex with anyone else.” All responses to these items were coded from 1: Strongly Disagree to 6: Strongly Agree, such that high index values represent a belief that sex partners can be identified as safe (polychoric *α* =.83, *M* = 2.12, SD = 1.25, *N* = 850).

#### 2.2.5. Pregnancy History

Previous pregnancies or attempts to get pregnant may affect the type of contraception utilized. A single item asked participants to indicate whether they or a partner had a previous pregnancy or tried to get pregnant during the past 12 months. Response options were (1) no or (2) yes.

### 2.3. Statistical Methods

Summary statistics and bar charts describe participants' reported use of contraception methods during their last sexual encounter. Tetrachoric correlations were then calculated between contraceptive methods. Respondents' contraceptive methods were then classified into one of six mutually exclusive categories: (1) condoms only, (2) condoms plus some other nonhormonal method, (3) Birth control pills or hormonal method (Depo-Provera) plus some method other than condoms, (4) condoms and hormonal method only, (5) All combinations not including condoms or hormonal methods, and (6) no method. To predict the contraceptive use classification, we use multinomial logistic regression [33] looking at the ability of respondents' gender, older age, type of partner at last sex event, the Select mediator, and pregnancy history to predict a respondent's membership in a multiple contraceptive typology. In the multinomial logistic regression model, the comparison condition was use of only condoms given our focus on dual and multiple contraception use.

## 3. Results

### 3.1. Respondent Characteristics

For this paper, we selected iMPPACS baseline survey respondents who reported on contraceptive use the last time they had vaginal sex (*N* = 850). African American adolescent females (*n *= 437) and males (*n *= 413) were recruited in four mid-sized cities: Syracuse, NY (*n *= 212, 25%), Providence, RI (*n* = 198, 23%), Macon, Ga (*n* = 226, 27%), and Columbia, SC (*n *= 214, 25%). Participants were 14 to 17 years old (*M *age = 15.4, SD = 1.1). The majority (93%) were living in their family's home, most often with their mother in the household (84%) and less frequently with their father in the home (20%). Most participants (73%) qualified for a free or reduced price lunch at school. Eighty-five percent were in high school (i.e., grades 9 through 12), 13% were in junior high (i.e., grades 7, 8), and 2% were not in school at the time of study enrollment. Fourteen percent of adolescent females (*n *= 61) reported one or more previous pregnancies and nine percent of adolescent males (*n *= 36) reported previously impregnating a partner.

### 3.2. Descriptive Statistics: Reported Contraceptive Use

**Figure 1:**
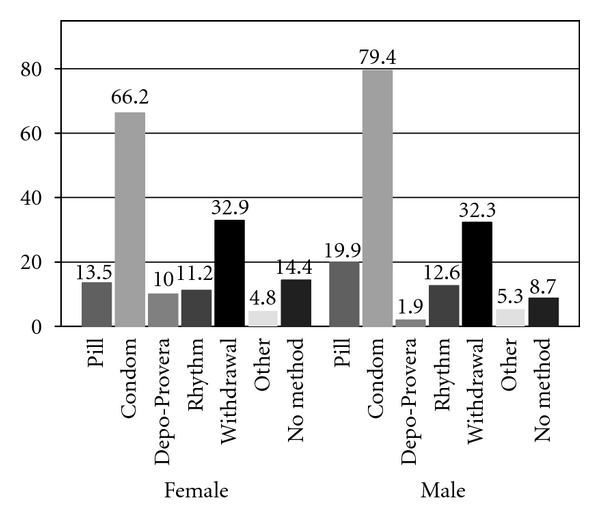
Prevalence of contraceptive method at last sex by gender (*N* = 850). Notes: percents sum to greater than 100% because of use of dual or multiple contraceptive methods.

Figure 1 shows the distribution of types of contraceptives used at the last vaginal sex event. For both males and females, the most common method was the male condom and the second most common method was withdrawal. The percentages sum to over 100% because of multiple contraceptive usage, so that the marginal distributions of each method give a distorted representation of contraceptive use. 

**Figure 2:**
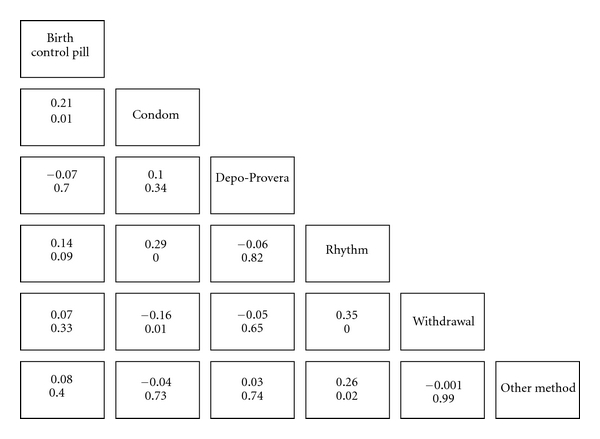
Tetrachoric correlations between contraceptive methods (*N *= 850). Note: significance level of correlation below coefficient.

Figure 2 shows the (tetrachoric) correlations between use of each method. The statistically significant dual methods were condoms/birth control pills, condoms/rhythm, condoms/withdrawal, withdrawal/rhythm, and other method/rhythm. However, even these significant correlations understate the variety of contraceptive use: 35 different contraceptive method combinations were reported by this adolescent sample (not counting the 12%, *n* = 99, who reported “no method used” at the last vaginal sex event). The most frequent combination was condoms/withdrawal (11%, *n* = 92), followed by condom/birth control pills (7%, *n* = 58), condom/birth control pills/withdrawal (4%, *n* = 31), condom/withdrawal/rhythm (3.5%, *n* = 30), and condom/rhythm (3.4%, *n* = 29). The most frequent method used alone was condoms (35%, *n* = 294), but single use of *any* of the other methods was rare: the next most recent single use was withdrawal (9%, *n* = 76) followed by birth control pill (<2%, *n* = 16). In other words, except for exclusive use of the male condom, virtually all contraceptive methods were dual or multiple in this sample.

### 3.3. Predicting Contraceptive Dual Use

To capture the heterogeneity in contraceptive use, the self-reported combinations of contraceptive uses were classified into six mutually exclusive types: (1) condoms only (“Condoms Only,” 36%), (2) Condoms plus some other non-hormonal method (“Condoms Plus,” 20%), (3) birth control pills or hormonal method (Depo-Provera) plus some method other than condoms (“Hormonal Plus,” 4%), (4) condoms and hormonal method only (“Both,” 18%), (5) all combinations not including condoms or hormonal methods (“Some Less Effective Method,” 12%), and (6) no method (“No Method,” 12%). Next, we predicted the typology using type of sex partner at last sex, the Select mediator, pregnancy history, age, and gender of the respondent in a multinomial logistic regression model. Because the focus is on dual/multiple use, the comparison classification was Condoms Only. These results are shown in Table 1. 

Results show that gender (Female) and the Select mediator are the primary predictors of the typology. Select is significantly positively associated with all methods relative to Condoms Only except for the Condoms Plus and Condom and Hormonal Only groups. In particular, it is a strong predictor of No Method used. Female gender is also predictive of Hormonal Plus and the two most risky categories, the Less Effective Method and No Method groups. For the most recent sex event, the type of sex partner and respondent age has little ability to predict the type of dual/multiple contraceptive use adjusting for the other variables in the equation. Previous pregnancies or pregnancy attempts were associated with decreased likelihood of all methods relative to the Condom Only group except Condoms Plus. 

## 4. Discussion

Current guidelines recommend the use of dual contraception methods to prevent unintended pregnancy and STD/HIV. Although the most common single contraception method used was the male latex condom (a strategy that affords both pregnancy and STD prevention), most adolescents did report the use of dual or multiple contraceptive methods. However, the types and combinations of contraceptive methods used provide varying degrees of reliable birth control and STD prevention. Similar to previous studies with African American adolescents [13, 14] only a minority of youth reported the use of hormonal birth control in combination with condoms, the dual method that provides the best protection against both pregnancy and STD/HIV. Instead, adolescents more frequently endorsed combining contraceptive practices that provide less effective protection against STD/HIV and unintended pregnancies (e.g., use of withdrawal and rhythm methods) and in some cases suggest improper use of a single method when used in conjunction with other contraceptive practices (e.g., use of condoms in combination with withdrawal) [34]. 

The perceived ability to select safe partners corroborates past research that has used survey, experimental, and qualitative methods [35–38] to show that adolescents (and adults) use informal rules (e.g., “heuristics”) to choose safe partners and to decide when to have sex [39], although such strategies are flawed and may result in negative health outcomes [40]. In this case, the use of this informal rule predicted the most risky contraceptive decisions. Individuals who believed they could identify safe partners were more likely to report using no contraception or using methods other than condoms. Consequently, adolescents who perceive that their partners present low STD risk may also forgo use of other effective contraceptive methods to prevent pregnancy. In contrast, the type of partner during the last sexual encounter was not predictive of the contraceptive method used. Thus, it appears that adolescents are using their partner *risk* heuristic rather than partner *type* heuristic when deciding about contraceptive methods. It may be that adolescents who believe they can select safe sexual partners make such estimations based upon a partner's individual characteristics (e.g., prior sexual experience) or other relationship factors (e.g., mutual monogamy) rather than focusing upon the partner type. 

Female gender also predicted the two most ineffective and risky categories as well: not using any contraceptive method and use of less effective contraceptive methods (e.g., withdrawal). Females were also more likely to report use of hormonal birth control as a primary contraceptive method (i.e., Hormonal Plus method type). Previous research highlights that young women tend to have less power in sexual relationships with their male partners which in turn may limit their ability to negotiate preventative sexual practices including condom use [41]. Thus, one may hypothesize that adolescent girls select contraceptive methods that maximize control (e.g., birth control pills), whereas their male partner has greater control of other contraceptive practices (e.g., condom use). Furthermore, one partner's use of a particular contraceptive method may moderate the perceived need for the other partner to use additional methods. For example, in a dyad where a young woman is using hormonal birth control, there may be less perceived need to also use condoms. In contrast to previous studies where age differences in contraceptive practices have been observed [8], age was not predictive of contraceptive methods in our study, perhaps because the age range in our sample was restricted. 

### 4.1. Limitations

Several limitations should be acknowledged. We do not have data regarding the extent to which the contraceptive method reported was consistently or properly used. For example, we did not assess condom use errors or the consistency by which birth control pills were taken. We also assessed only the most popular methods and did not assess methods that are less commonly used by adolescents (e.g., vaginal ring). Thus, actual rates of hormonal birth control usage may be slightly higher than reported by participants. Further, the contraception method measure focused on the methods used to prevent pregnancy during the last sexual event, so reported methods may not fully reflect participants' typical contraceptive practices across sexual encounters. Additionally, the participants were recruited from four medium-sized cities; results may not generalize to youth from larger cities or rural areas.

### 4.2. Conclusions

Our findings highlight the need to further understand how adolescents select contraceptive methods. Because contraception is used within the context of a dyad, studies should examine how adolescents communicate and negotiate the use of individual, dual, and multiple contraceptive practices with their partners. Knowledge of contraceptive practices could then facilitate the development of appropriate intervention messages to prevent both unintended pregnancies and STD/HIV among African American adolescents. Interventions may benefit from inclusion of material to improve adolescents' ability to communicate with partners about contraceptive methods and explain the health risks posed by incorrect appraisal of a partner's risk. Such prevention programs could also target the power differential within sexual partnerships and provide adolescent females with strategies to negotiate safer sexual practices. Ultimately, comprehensive sexual health services and interventions will facilitate adolescents' ability to have healthy sexual relationships and prevent negative health outcomes.

## Figures and Tables

**Table 1:** Results of predicting dual/multiple contraceptive use typology (*N* = 850).

Predictor Variable	Contraceptive use category				
	Condoms Plus	Hormonal Plus	Condom and Hormonal Only	Some Less Effective Method	No Method
Select safe partners	1.01 (.85 – 1.20)	***1.50 ***(1.15– 1.96)	1.16 (.98–1.37)	***1.23 ***(1.01–1.49)	***1.25 ***(1.03–1.51)

Someone you just met or casual partner	.64 (.31–1.33)	1.46 (.47–4.51)	.72 (.35–1.49)	1.13 (.49–2.60)	1.77 (.83–3.87)

Someone you knew well but not a steady partner	.76 (.48–1.19)	.55 (.20 –1.55)	***.46*** (.27–.78)	.98 (.57–1.72)	.97 (.55–1.74)

Female	1.01 (.67 –1.50)	***2.23 ***(1.02–4.89)	1.18 (.77–1.80)	***2.47 ***(1.49–4.10)	***2.37 ***(1.41–3.97)

16-17 years old at baseline	.69 (.47 –1.01)	***3.76 ***(1.64–8.65)	1.39 (.93–2.07)	1.15 (.72–1.82)	.94 (.58–1.51)

Pregnancy attempt or pregnancy in past 12 months	.45 (.18–1.13)	***.18 ***(.06 –.55)	***.40*** (.16 –.96)	***.35*** (.13–.90)	***.11*** (.05–.24)

Notes: entries are odds ratios relative to the omitted group (Condoms Only). Bold, italic coefficients are significant at  .05 level or less. Confidence intervals of odds ratio in parentheses.
